# Exploring the association between immune-inflammation index and carotid plaque formation: a cross-sectional study in a large Chinese health screening population

**DOI:** 10.3389/fendo.2025.1732824

**Published:** 2025-12-05

**Authors:** Jinxiao Sun, Xialing Zhang, Meng Yang, Shuo Yang, Hua Zeng

**Affiliations:** 1 Department of Ultrasound, Guangzhou 11th People’s Hospital, Guangzhou, Guangdong, China; 2 Department of Ultrasound, Guangzhou Cadre and Talent Health Management Center, Guangdong, China

**Keywords:** carotid plaque, aggregate index of systemic inflammation, systemic inflammation response index, systemic immune-inflammation index, atherosclerosis, cardiovascular risk assessment

## Abstract

**Purpose:**

Cardiovascular disease remains a major public health concern and is closely associated with carotid atherosclerosis, a lipid-driven inflammatory condition. Composite inflammatory indices, including the systemic immune-inflammation index (SII), systemic inflammation response index (SIRI), and aggregate index of systemic inflammation (AISI), have shown promise in cardiovascular risk assessment; however, their comparative predictive value for carotid plaque formation has not been adequately validated in large Asian populations. This study investigated the associations between these inflammatory indices and carotid plaque presence in a large-scale Chinese health screening cohort.

**Patients and methods:**

This cross-sectional study analyzed 9,503 adults (mean age 51.6 ± 9.5 years; 50.8% male) who underwent comprehensive health examinations at Guangzhou 11th People’s Hospital between January 2018 and December 2022. Inflammatory indices were calculated from complete blood counts: SII = (neutrophils × platelets)/lymphocytes, SIRI = (neutrophils × monocytes)/lymphocytes, and AISI = (neutrophils × platelets × monocytes)/lymphocytes. Carotid plaques were identified using standardized ultrasonography according to Mannheim Consensus criteria. Best subset regression with rigorous 10-fold cross-validation identified optimal prediction models from 4,095 potential combinations. The cohort was divided into training (70%, n=6,652) and validation (30%, n=2,851) sets for model development and internal validation.

**Results:**

Carotid plaque prevalence was 29.2%. All inflammatory indices were significantly higher in participants with plaques: SIRI (0.78 ± 0.50 vs. 0.63 ± 0.36, P<0.001), AISI (2.04 ± 1.43 vs. 1.57 ± 0.99, P<0.001), and SII (5.28 ± 2.66 vs. 4.32 ± 1.88, P<0.001). Among 89 models without multicollinearity, the optimal four-variable model included age (OR = 1.028, 95% CI: 1.020–1.036), fasting glucose (OR = 1.799, 95% CI: 1.657–1.952), AISI (OR = 2.277, 95% CI: 2.072–2.502), and diabetes mellitus (OR = 3.234, 95% CI: 2.727–3.836). This model achieved superior validation performance (AUC = 0.744) compared with models incorporating SIRI (AUC = 0.739) or traditional risk factors alone (AUC = 0.731). At the optimal threshold (0.32), the model demonstrated 71.5% sensitivity, 68.9% specificity, and 69.4% accuracy. Calibration was excellent (Hosmer–Lemeshow P = 0.511; Brier score=0.198).

**Conclusion:**

AISI emerged as the most robust inflammatory biomarker for carotid plaque prediction among composite indices, suggesting its superior ability to capture the complex interplay between neutrophils, monocytes, platelets, and lymphocytes in atherosclerosis. The developed four-variable model combining AISI with traditional risk factors provides a clinically feasible tool for carotid atherosclerosis risk stratification in Chinese populations, potentially enhancing early detection and preventive interventions.

## Introduction

Cardiovascular disease (CVD) remains a major global public health challenge and is closely linked to carotid atherosclerosis, a lipid-driven, multifactorial inflammatory condition (1, 2). Carotid atherosclerosis is characterized by progressive thickening of the carotid intima–media (cIMT) and the formation of atherosclerotic plaques within the arterial wall (3). Inflammation is a pivotal pathophysiological mechanism underlying atherosclerosis progression and plaque destabilization (4). The inflammatory cascade involves diverse cellular components—including neutrophils, lymphocytes, monocytes, and platelets—each contributing distinct pathological mechanisms that collectively drive vascular inflammation and endothelial dysfunction (5). However, traditional inflammatory biomarkers such as C-reactive protein (CRP) and erythrocyte sedimentation rate (ESR) are limited by their nonspecific nature and their inability to capture the complex interplay among different immune cell populations (6, 7).

Recent advances in understanding cardiovascular inflammation have led to the development of composite inflammatory indices—including the systemic immune-inflammation index (SII), systemic inflammation response index (SIRI), and aggregate index of systemic inflammation (AISI)—which integrate multiple immune cell populations to better reflect systemic inflammatory burden (8, 9). SII has been associated with coronary artery disease severity and independently predicts major adverse cardiovascular events (10, 11), while SIRI correlates with acute coronary syndrome occurrence and stroke risk in older populations (12). AISI has emerged as a predictor of adverse outcomes in cardiovascular patients (13). These indices also show associations with carotid artery stenosis severity, peripheral arterial disease, and subclinical atherosclerosis measures (14, 15), suggesting their potential as robust biomarkers for cardiovascular risk stratification in atherosclerotic disease management.

Although composite inflammatory indices have demonstrated clinical value in atherosclerotic disease assessment, current research presents several important limitations (16). Most existing studies use modest sample sizes, limiting statistical power and generalizability. Large-scale validation in Asian populations remains limited despite potential ethnic differences in inflammatory profiles (17). Additionally, systematic comparisons of different composite inflammatory indices using rigorous methodologies are lacking, and many studies employ inadequate model validation procedures (18). These limitations underscore the need for well-designed studies with appropriate sample sizes and rigorous validation.

To address these gaps, the present study investigated the association between composite inflammatory indices (SII, SIRI, and AISI) and carotid plaque formation in a large Chinese health screening population. Using systematic best subset regression analysis and rigorous internal validation, we aimed to identify the optimal combination of inflammatory and clinical parameters for predicting carotid plaque presence, thereby developing a clinically simple, feasible, and reliable predictive model for carotid atherosclerosis risk assessment.

## Materials and methods

### Study design and population

This study was conducted at Guangzhou 11th People’s Hospital between January 2018 and December 2022. Of 23,173 adults who underwent comprehensive health examinations, 9,503 participants were included after applying strict inclusion and exclusion criteria. Inclusion criteria were: (1) age ≥18 years; (2) complete carotid ultrasonography; (3) complete blood count and biochemical parameters; and (4) complete demographic and clinical data. Exclusion criteria were: (1) acute or chronic infectious diseases within the previous 3 months; (2) history of malignancy or current oncologic treatment; (3) severe liver or kidney dysfunction; (4) autoimmune diseases or immunosuppressive therapy; (5) pregnancy; (6) incomplete or poor-quality carotid ultrasonography images; (7) incomplete clinical or laboratory data; and (8) any condition likely to significantly affect inflammatory marker levels.

The study was conducted in accordance with the Declaration of Helsinki and was approved by the Ethics and Research Committee of Guangzhou 11th People’s Hospital. All participant data were de-identified and anonymized to ensure confidentiality, with strict security measures implemented for data handling. Data were used exclusively for scientific research purposes. Given the retrospective design and use of anonymized data, the requirement for informed consent was waived by the ethics committee.

### Demographic and clinical variables

Standardized questionnaires collected demographic information, including age, sex, anthropometric measurements, medical history, and lifestyle factors. Body mass index was calculated as weight (kg) divided by height squared (m²). Clinical conditions were defined as follows: Hypertension: systolic BP ≥140 mmHg and/or diastolic BP ≥90 mmHg on repeated measurements, or current antihypertensive medication use. Diabetes mellitus: fasting glucose ≥7.0 mmol/L, HbA1c ≥6.5%, or antidiabetic medication use. Dyslipidemia: according to the 2016 Chinese Guidelines, defined as total cholesterol ≥6.2 mmol/L, LDL-C ≥4.1 mmol/L, HDL-C <1.0 mmol/L (males) or <1.3 mmol/L (females), or triglycerides ≥2.3 mmol/L. Current smoking: ≥1 cigarette daily for >6 months. Alcohol consumption: regular intake ≥once weekly for >6 months.

Inflammatory Index Calculations:


SII=(neutrophil×platelet)/lymphocyte count

SIRI=(neutrophil×monocyte)/lymphocyte count

AISI=(neutrophil×platelet×monocyte)/lymphocyte count

### Carotid ultrasonography

Carotid ultrasonography was performed by certified sonographers (≥5 years of experience) using a standardized protocol with a SonoScape S60 ultrasound system equipped with a 10-MHz linear transducer (SonoScape Medical Corp., Shenzhen, China). Participants were positioned supine with the neck extended, and bilateral carotid arteries were systematically examined, including the common carotid artery, bifurcation, and internal and external carotid arteries in longitudinal and transverse planes. Carotid plaque was defined according to the Mannheim Carotid Intima-Media Thickness and Plaque Consensus as a focal structure encroaching on the arterial lumen ≥1.5 mm, localized thickening ≥50% of the surrounding intima–media thickness, or thickness >1.5 mm measured from the media–adventitia to the intima–lumen interface. Ten percent of examinations were randomly re-evaluated by a second experienced sonographer blinded to clinical data. Interobserver agreement for plaque detection was excellent (κ=0.91, 95% CI: 0.86–0.96).

### Statistical analysis

Statistical analyses were performed using R version 4.2.0 (R Foundation for Statistical Computing, Vienna, Austria) and SPSS 26.0 (IBM Corporation, Armonk, NY, USA). Univariate analyses compared variables between groups using appropriate statistical tests: independent t-tests or Mann–Whitney U tests for continuous variables, and chi-square or Fisher’s exact tests for categorical variables. Variables with P<0.05 were retained for multivariable modeling.

The cohort was randomly divided into training (70%, n=6,652) and validation (30%, n=2,851) sets using stratified randomization (seed=42) to maintain a balanced outcome distribution. Best subset regression systematically evaluated all combinations of significant variables. Each model underwent 10-fold cross-validation repeated five times to calculate mean AUC. Variance inflation factors were assessed to exclude models with multicollinearity (VIF ≥10).

A two-stage selection process was employed: (1) models with cross-validated AUC in the top 10th percentile were identified as high-performing candidates; and (2) among these, models with the fewest variables were selected and tested in the validation cohort. The model achieving the highest validation AUC was designated as the final prediction model. Model discrimination was evaluated using ROC analysis, with AUC and 95% confidence intervals calculated by the DeLong method. Calibration was assessed using the Hosmer–Lemeshow test and calibration plots. Performance metrics included sensitivity, specificity, positive and negative predictive values, and accuracy at the optimal cut-point determined by the Youden Index.

We employed best subset regression combined with logistic regression modeling for optimal predictor identification. Best subset regression systematically evaluates all possible predictor combinations to identify the optimal model, providing an exhaustive search superior to stepwise methods that may miss optimal variable combinations. This approach has been successfully applied in cancer prognosis prediction (19) and cardiovascular risk assessment (20). Following variable selection, we utilized multivariable logistic regression to estimate the probability of carotid plaque presence. Logistic regression is particularly suitable for clinical prediction models due to its interpretability, clinically meaningful odds ratios, and ability to generate individual probability scores for risk stratification (21). This combined approach ensures optimal predictor identification while maintaining model interpretability.

All statistical tests were two-sided, with significance set at P<0.05. Results were reported as odds ratios with 95% confidence intervals.

## Result

### Study population characteristics

A total of 9,503 participants were included in the final analysis (mean age 51.6 ± 9.5 years; 50.8% male), with an overall carotid plaque prevalence of 29.2%. The cohort was randomly divided into training (n=6,652; 70%) and validation (n=2,851; 30%) sets. Baseline characteristics were well balanced between cohorts, with no significant differences in age (P = 0.071), sex distribution (P = 0.956), carotid plaque prevalence (P = 0.924), or any clinical variables (all P>0.05). Comorbidities and lifestyle factors were also similarly distributed, confirming successful randomization (**Table 1**).

**Table 1:** Baseline characteristics of training and validation cohorts.

Variable	Total (n=9,503)	Training cohort (n=6,652)	Validation cohort (n=2,851)	P- value
Age (years)	51.6 ± 9.5	51.4 ± 9.5	51.8 ± 9.6	0.071
Male sex, n (%)	4,832 (50.8)	3,384 (50.9)	1,448 (50.8)	0.956
BMI (kg/m²)	24.02 ± 3.06	24.01 ± 3.05	24.05 ± 3.08	0.578
Fasting glucose (mmol/L)	5.42 ± 1.01	5.41 ± 1.00	5.44 ± 1.03	0.175
Triglycerides (mmol/L)	1.50 ± 1.31	1.49 ± 1.30	1.52 ± 1.33	0.295
Total cholesterol (mmol/L)	5.22 ± 0.99	5.21 ± 0.98	5.24 ± 1.01	0.189
HDL-C (mmol/L)	1.53 ± 0.37	1.54 ± 0.37	1.52 ± 0.38	0.087
LDL-C (mmol/L)	2.97 ± 0.83	2.97 ± 0.82	2.98 ± 0.85	0.682
Inflammatory Indices				
SIRI	0.67 ± 0.41	0.66 ± 0.40	0.69 ± 0.42	0.001
AISI	1.71 ± 1.15	1.70 ± 1.14	1.74 ± 1.18	0.110
SII	4.63 ± 2.18	4.61 ± 2.16	4.67 ± 2.23	0.228
Comorbidities, n(%)				
Hypertension, n(%)	1,979 (20.8)	1,386 (20.8)	593 (20.8)	0.978
Diabetes mellitus, n(%)	1,030 (10.8)	721 (10.8)	309 (10.8)	0.996
Dyslipidemia, n(%)	5,796 (61.0)	4,060 (61.0)	1,736 (60.9)	0.890
Lifestyle Factors, n(%)				
Current smoking, n(%)	1,519 (16.0)	1,063 (16.0)	456 (16.0)	0.997
Alcohol consumption	1,700 (17.9)	1,191 (17.9)	509 (17.9)	0.987
Carotid plaque, n (%))	2,779 (29.2	1,947 (29.3)	832 (29.2)	0.924

Data are presented as mean ± SD for continuous variables and n (%) for categorical variables. BMI, body mass index; HDL-C, high-density lipoprotein cholesterol; LDL-C, low-density lipoprotein cholesterol; SIRI, systemic inflammation response index; AISI, aggregate index of systemic inflammation; SII, systemic immune-inflammation index.

### Univariate analysis of carotid plaque–associated factors

Twelve variables showed significant associations with carotid plaque presence. Participants with plaque were older (52.58 ± 10.16 vs. 51.08 ± 9.13 years, P<0.001) and had higher fasting glucose (5.69 ± 1.28 vs. 5.28 ± 0.82 mmol/L, P<0.001) and triglycerides (1.58 ± 1.25 vs. 1.47 ± 1.33 mmol/L, P<0.001), but lower HDL-C levels (1.49 ± 0.35 vs. 1.55 ± 0.38 mmol/L, P<0.001). All inflammatory indices were significantly elevated in the plaque group: SIRI (0.78 ± 0.50 vs. 0.63 ± 0.36, P<0.001), AISI (2.04 ± 1.43 vs. 1.57 ± 0.99, P<0.001), and SII (5.28 ± 2.66 vs. 4.32 ± 1.88, P<0.001). Diabetes mellitus showed the strongest categorical association (χ²=411.970, P<0.001), followed by hypertension, smoking, and alcohol consumption (all P<0.001). Sex, BMI, total cholesterol, and LDL-C showed no significant associations (**Table 2**).

**Table 2:** Univariate analysis of factors associated with carotid plaque.

Variable	No plaque (n=6,724)	Plaque present (n=2,779)	est statistic (t/χ²)	P-value
Age (years)	51.08 ± 9.13	52.58 ± 10.16	-6.733	<0.001
Fasting glucose (mmol/L)	5.28 ± 0.82	5.69 ± 1.28	-15.465	<0.001
BMI (kg/m²)	23.99 ± 3.06	24.11 ± 3.05	-1.769	0.077
Triglycerides (mmol/L)	1.47 ± 1.33	1.58 ± 1.25	-3.947	<0.001
Total cholesterol (mmol/L)	5.23 ± 0.95	5.19 ± 1.07	1.562	0.118
HDL-C (mmol/L)	1.55 ± 0.38	1.49 ± 0.35	8.290	<0.001
LDL-C (mmol/L)	2.98 ± 0.80	2.96 ± 0.89	0.945	0.345
SIRI	0.63 ± 0.36	0.78 ± 0.50	-14.461	<0.001
AISI	1.57 ± 0.99	2.04 ± 1.43	-15.866	<0.001
SII	4.32 ± 1.88	5.28 ± 2.66	-17.439	<0.001
Male sex	3,427 (50.9)	1,405 (50.6)	0.132	0.716
Hypertension			324.335	<0.001
Present	1,076 (54.4)	903 (45.6)		
Absent	5,648 (75.1)	1,876 (24.9)		
Current smoking			122.414	<0.001
Present	895 (58.9)	624 (41.1)		
Absent	5,829 (73.0)	2,155 (27.0)		
Alcohol consumption			199.195	<0.001
Present	963 (56.6)	737 (43.4)		
Absent	5,761 (73.8)	2,042 (26.2)		
Diabetes mellitus			411.970	<0.001
Present	449 (43.6)	581 (56.4)		
Absent	6,275 (74.1)	2,198 (25.9)		
Dyslipidemia			8.770	0.003
Present	4,037 (69.7)	1,759 (30.3)		
Absent)	2,687 (72.5)	1,020 (27.5		

Data are presented as mean ± SD for continuous variables and n (%) for categorical variables. P-values were calculated using independent-samples t-test for continuous variables and chi-square test for categorical variables.

### Best subset regression analysis and model selection

To identify the optimal prediction model, we performed comprehensive best subset regression on the 12 significant univariate variables in the training cohort. This exhaustive search evaluated all possible variable combinations, yielding 4,095 potential models. Each model underwent rigorous 10-fold cross-validation repeated five times to ensure robust performance estimates.

After excluding models with variance inflation factors ≥10 to control for multicollinearity, 89 models remained for evaluation. Models with mean cross-validated AUC in the top 10th percentile (AUC ≥0.730) were designated as high-performing candidates.

Four candidate models emerged, each comprising four variables, representing an optimal balance between predictive performance and parsimony. Model 1 (Age + Fasting glucose + SIRI + Diabetes) achieved the highest training AUC of 0.742 ± 0.018, followed by Model 2 (Age + Fasting glucose + AISI + Diabetes) with an AUC of 0.738 ± 0.019. Model 3 (Age + Fasting glucose + Hypertension + Diabetes) and Model 4 (Fasting glucose + SIRI + Hypertension + Diabetes) achieved AUCs of 0.735 ± 0.017 and 0.733 ± 0.020, respectively. All models demonstrated excellent multicollinearity control, with maximum VIF values ranging from 2.15 to 2.91 (**Table 3**).

**Table 3:** Best subset regression analysis results in training cohort.

Model	Variable combination	Mean AUC	SD	Max VIF	AUC range
Model 1	Age + Fasting glucose + SIRI + Diabetes	0.742	0.018	2.84	0.698-0.781
Model 2	Age + Fasting glucose + AISI + Diabetes	0.738	0.019	2.91	0.694-0.778
Model 3	Age + Fasting glucose + Hypertension +Diabetes	0.735	0.017	2.15	0.701-0.772
Model 4	Fasting glucose + SIRI + Hypertension + Diabetes	0.733	0.020	2.67	0.688-0.775

Results based on 10-fold cross-validation repeated 5 times. AUC threshold for top 10% models = 0.730. All models had VIF < 10, indicating adequate control of multicollinearity. AUC, area under the curve; SD, standard deviation; VIF, variance inflation factor; SIRI, systemic inflammation response index; AISI, aggregate index of systemic inflammation.

### Internal validation and final model selection

The four candidate models were subsequently evaluated in the independent validation cohort to assess generalizability. Model 2 achieved the highest validation AUC of 0.744, outperforming Model 1 (0.739), Model 3 (0.731), and Model 4 (0.728). At the optimal cut-point determined by the Youden Index (probability threshold=0.32), Model 2 demonstrated balanced performance metrics in the validation cohort: sensitivity 71.5%, specificity 68.9%, accuracy 69.4%, positive predictive value 45.8%, and negative predictive value 86.5% (**Table 4**).

**Table 4:** External validation performance of candidate models.

Model	Training AUC	Validation AUC	Sensitivity (%)	Specificity (%)	Accuracy (%)	PPV (%)	NPV (%)
Model 1	0.742	0.739	70.2	68.1	68.6	44.5	85.6
Model 2	0.738	0.744	71.5	68.9	69.4	45.8	86.5
Model 3	0.735	0.731	68.5	67.2	67.5	42.5	84.7
Model 4	0.733	0.728	67.8	66.5	66.8	41.8	84.2

Model 2 achieved the highest validation AUC and was selected as the final model. Performance metrics calculated using optimal cut-point determined by Youden Index. PPV, positive predictive value; NPV, negative predictive value.

The final logistic regression model incorporated four variables: age, fasting glucose, AISI, and diabetes mellitus. All variables were highly significantly associated with carotid plaque presence (all P<0.001). Diabetes mellitus was the strongest predictor (OR = 3.234, 95% CI: 2.727–3.836), followed by AISI (OR = 2.277, 95% CI: 2.072–2.502), fasting glucose (OR = 1.799, 95% CI: 1.657–1.952), and age (OR = 1.028, 95% CI: 1.020–1.036) (**Table 5**).

**Table 5:** Final prediction model: logistic regression coefficients.

Variable	Coefficient(β)	Standard Error	Wald χ²	P-value	OR	95% CI
Constant	-4.156	0.312	177.23	<0.001	–	–
Age(years)	0.028	0.004	49.84	<0.001	1.028	1.020-1.036
Fasting glucose (mmol/L)	0.587	0.042	195.66	<0.001	1.799	1.657-1.952
AISI	0.823	0.048	294.01	<0.001	2.277	2.072-2.502
Diabetes mellitus	1.174	0.087	182.15	<0.001	3.234	2.727-3.836

Model equation: Logit(P) = -4.156 + 0.028×Age + 0.587×Fasting glucose + 0.823×AISI +1.174×Diabetes, where P is the probability of carotid plaque presence. OR, odds ratio; CI, confidence interval; AISI, aggregate index of systemic inflammation.

### Model performance assessment

**Figure 1:**
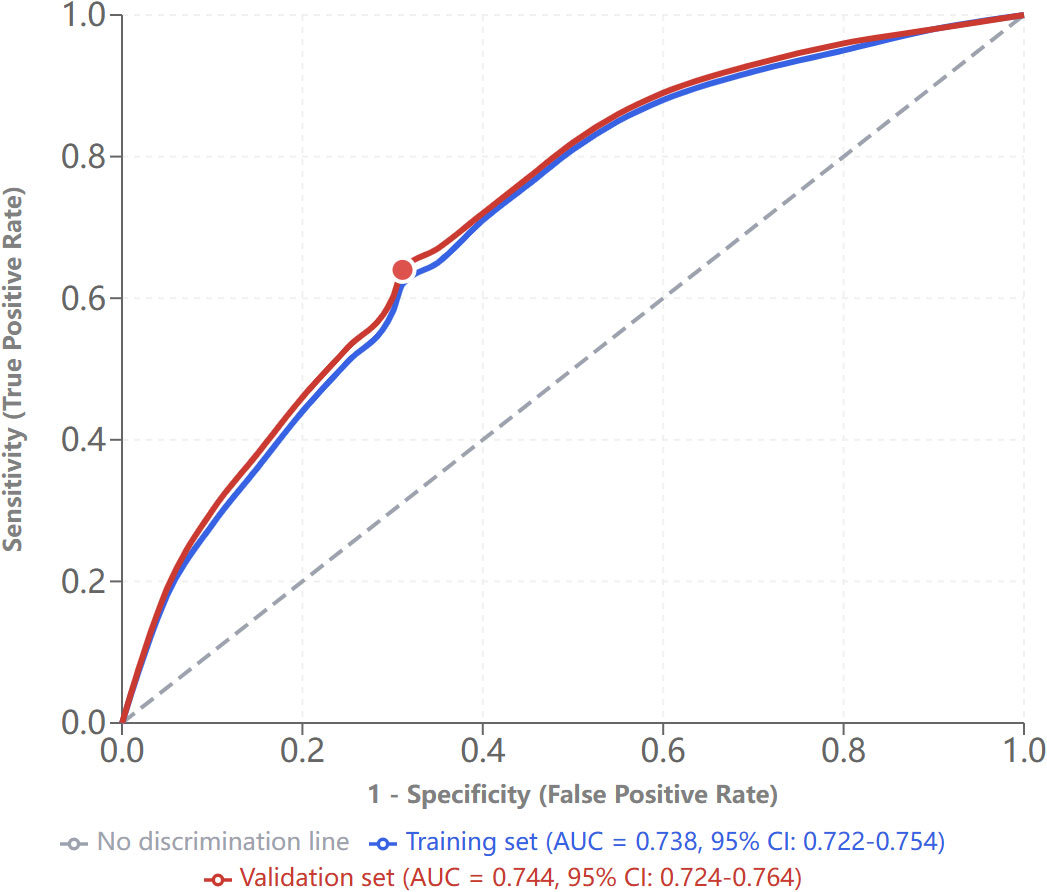
ROC curves for carotid plaque prediction model.

**Figure 2:**
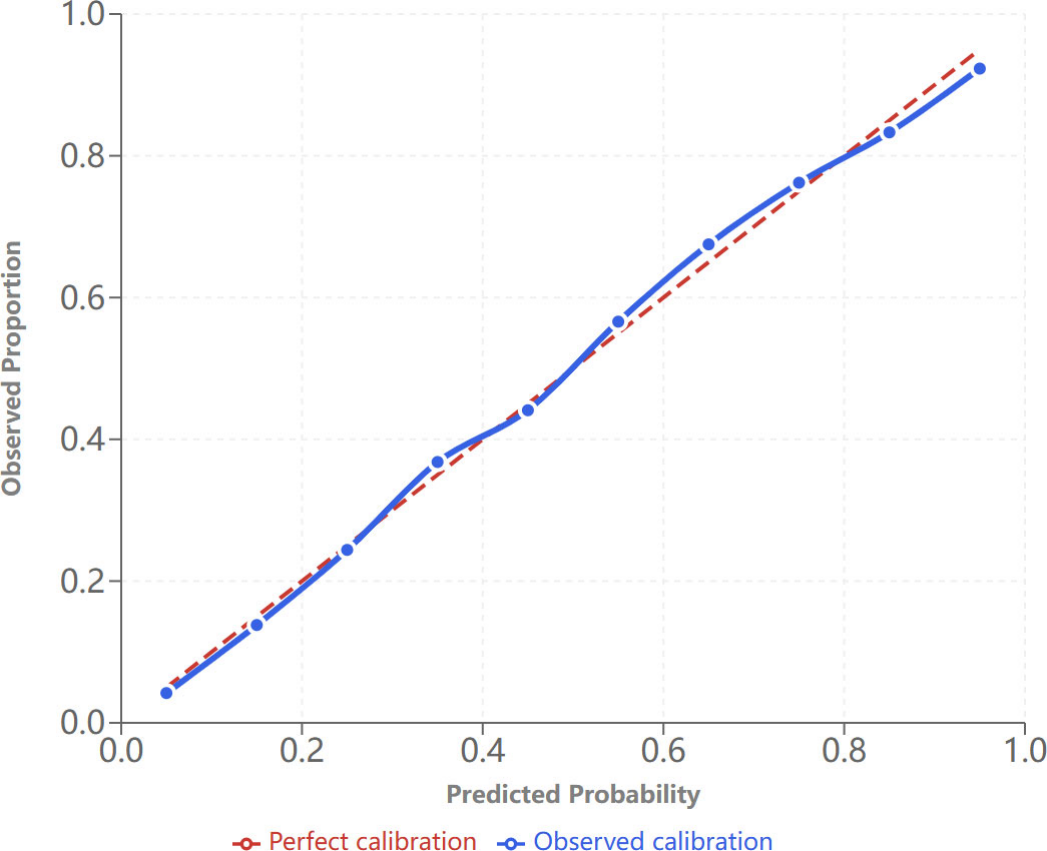
Calibration plot for the final prediction model.

The final model demonstrated excellent discrimination, with AUCs of 0.738 in the training cohort and 0.744 in the validation cohort (P = 0.672, DeLong test) (**Figure 1**). Calibration was excellent in both cohorts (Hosmer–Lemeshow P = 0.345 for training; P = 0.511 for validation), with calibration curves closely following the ideal line. The Brier score was 0.198, with a calibration slope of 0.96 and intercept of 0.02, confirming optimal calibration performance (**Figure 2**).

## Discussion

This large-scale investigation of 9,503 Chinese health screening participants provides robust evidence establishing AISI as the superior inflammatory biomarker for carotid plaque prediction among currently available composite indices. We found that SII, SIRI, and AISI were all significantly associated with carotid plaque formation. Through rigorous best subset regression evaluating 4,095 potential model combinations, our final four-variable model—incorporating age, fasting glucose, AISI, and diabetes mellitus—achieved an AUC of 0.744 in an independent validation cohort, demonstrating strong discriminative ability. This finding not only validates the pivotal role of systemic inflammation in carotid atherosclerosis development but also offers a clinically practical tool for risk stratification in primary prevention settings.

The pathological significance of carotid plaque formation extends beyond simple arterial narrowing. Carotid atherosclerotic disease represents a progressive cerebrovascular threat, with consequences ranging from transient ischemic attacks to catastrophic stroke, wherein vascular risk escalates with increasing luminal narrowing and plaque instability (1–3). Early detection and aggressive management of modifiable risk factors are critical to preventing severe cerebrovascular complications. Recognized risk factors include traditional cardiovascular determinants—hypertension, dyslipidemia, diabetes mellitus, smoking, and advanced age—alongside emerging inflammatory biomarkers that capture the systemic inflammatory burden underlying atherosclerotic progression (22–24).

Building on this foundation, current evidence robustly supports systemic inflammation as an integral component of atherosclerotic pathogenesis, wherein persistent low-grade inflammation promotes disease progression (25). Consistent with prior investigations, the present study identified significant associations between all three composite inflammatory indices—SII, SIRI, and AISI—and carotid plaque presence. All indices were significantly higher in participants with plaques: SIRI (0.78 ± 0.50 vs. 0.63 ± 0.36, P<0.001), AISI (2.04 ± 1.43 vs. 1.57 ± 0.99, P<0.001), and SII (5.28 ± 2.66 vs. 4.32 ± 1.88, P<0.001). These findings suggest that individuals with elevated systemic inflammation profiles face a substantially higher risk of developing carotid plaques. While low-density lipoprotein cholesterol is a principal driver of atherosclerotic pathology, atherosclerosis is fundamentally an inflammatory disease characterized by dynamic interactions among multiple cellular populations that collectively orchestrate disease progression.

Among these indices, AISI demonstrated superior predictive performance, a finding with important mechanistic and clinical implications. In a cohort of 830 patients with type 2 diabetes, Wang et al. reported that AISI mediated 10.9% of the association between metabolic dysfunction–associated fatty liver disease and subclinical carotid atherosclerosis—exceeding SII’s mediation effect of 7.8%—suggesting AISI’s superior capacity to capture inflammatory–metabolic interactions (24). Our observed effect size for AISI (OR = 2.277, 95% CI: 2.072–2.502) is consistent with the meta-analysis by Zhao et al., which reported a pooled hazard ratio of 2.36 for SII across 15,832 patients with coronary artery disease (16). Importantly, our study establishes AISI’s superiority through direct, within-cohort comparison of all three indices using identical methodology, thereby minimizing interstudy heterogeneity and extending applicability to an asymptomatic screening population—an advance with meaningful implications for primary prevention in apparently healthy individuals.

This pathophysiological superiority of AISI derives from its unique integration of four key cellular mediators of atherosclerotic inflammation. Whereas SII captures platelet–neutrophil–lymphocyte dynamics and SIRI incorporates monocyte–neutrophil–lymphocyte interactions, AISI integrates all four cell types, providing a more complete assessment of systemic inflammatory burden. Neutrophils contribute to plaque destabilization through the release of proteolytic enzymes and reactive oxygen species, degrading extracellular matrix and amplifying local inflammation (26, 27). Monocytes, recruited through chemotactic gradients, differentiate into macrophages and foam cells, secreting proinflammatory cytokines and matrix metalloproteinases that promote plaque vulnerability (28). Platelets further amplify inflammation and precipitate thrombotic events (5, 23). Concurrently, lymphocyte depletion reflects impaired adaptive immune regulation, permitting unchecked innate immune activation. AISI’s capacity to capture these interdependent pathways—the combined dysregulation of innate immunity (elevated neutrophils, monocytes, platelets) and adaptive immune dysfunction (reduced lymphocytes)—mechanistically explains its superior predictive performance.

The clinical translation of these findings is strengthened by our methodological approach. Unlike prior investigations that employed stepwise regression or predetermined variable selection, our exhaustive evaluation of all possible variable combinations via best subset regression, combined with rigorous 10-fold cross-validation repeated five times, ensured optimal model selection while controlling for multicollinearity. The maximum variance inflation factor of 2.91 across all examined models was well below the conventional threshold of 10, confirming appropriate model specification. The model’s excellent calibration—Hosmer–Lemeshow P = 0.511, Brier score 0.198, calibration slope 0.96, and intercept 0.02—indicates that predicted probabilities closely reflect observed risks across the full spectrum, addressing a critical gap in biomarker validation often overlooked in the clinical literature (29–31). The notable stability between training and validation performance (AUC 0.738 vs. 0.744, P = 0.672) demonstrates robust generalizability and substantially reduces concerns regarding overfitting.

At the optimal probability threshold of 0.32, the model showed balanced performance with 71.5% sensitivity, 68.9% specificity, and a notably high negative predictive value of 86.5% in the independent validation cohort. This high negative predictive value provides meaningful clinical utility by substantially reducing the likelihood of advanced carotid disease in individuals below the threshold and enabling more efficient resource allocation by reducing unnecessary imaging in low-risk populations. Among model components, diabetes mellitus emerged as the strongest predictor (OR = 3.234, 95% CI: 2.727–3.836), consistent with established mechanisms whereby hyperglycemia accelerates atherosclerotic progression through advanced glycation end-product formation, enhanced oxidative stress, and amplified inflammatory signaling (32–34). The model’s simplicity—requiring only four readily available variables (age, fasting glucose, AISI from a routine complete blood count, and diabetes status)—substantially enhances its translational potential for implementation in resource-limited settings and routine primary care.

Despite these strengths, several limitations warrant consideration. The cross-sectional design precludes causal inference regarding temporal relationships between inflammatory elevation and plaque development. Single-center recruitment, while ensuring standardized protocols, may limit generalizability to populations with different genetic backgrounds or environmental exposures. Additionally, single time-point inflammatory assessment cannot capture dynamic changes that might provide additional prognostic information, and potential unmeasured confounders—including medication effects or subclinical infections—could influence inflammatory marker levels. Future prospective studies with serial AISI monitoring are needed to establish temporal relationships, evaluate whether AISI-guided interventions reduce plaque progression, and validate the model across diverse ethnic populations.

In conclusion, this study establishes AISI as the most robust composite inflammatory biomarker for carotid plaque prediction through comprehensive integration of neutrophil, monocyte, platelet, and lymphocyte dynamics. The rigorously validated four-variable prediction model offers a practical tool for risk stratification in Chinese populations, potentially enabling earlier preventive interventions and improved cardiovascular outcomes. These findings warrant external validation in diverse populations and prospective evaluation of AISI-guided strategies in clinical practice.

## Data Availability

The raw data supporting the conclusions of this article will be made available by the authors, without undue reservation.
